# Subsequent ovarian yolk sac tumor after operation of ovarian mature teratoma: a case report and review of the literature

**DOI:** 10.3389/fonc.2023.1327724

**Published:** 2024-01-17

**Authors:** Shuqing Li, Juan Peng, Yajun Zhang, Dongxia Liu, Lei Li, Manman Nai

**Affiliations:** ^1^ The Department of Obstetrics and Gynecology, The Third Affiliated Hospital of Zhengzhou University, Zhengzhou, China; ^2^ Zhengzhou Key Laboratory of Endometrial Disease Prevention and Treatment, Zhengzhou Science and Technology Bureau, Zhengzhou, China

**Keywords:** ovary, teratoma, yolk sac tumor, case report, literature review

## Abstract

Ovarian mature teratoma represents a benign ovarian tumor, while ovarian yolk sac tumor (YST, endodermal sinus tumor) is a rare malignant tumor predominantly affecting young women, often associated with a grim prognosis post-metastasis. Both ovarian mature teratoma and ovarian YST are germ cell tumors. There are few studies on the correlation between ovarian YST and mature teratoma. Recurrence or malignant transformation may occur following the surgical intervention for ovarian mature teratoma. However, the occurrence of YST subsequent to such procedures is notably rare. In this investigation, we reported a case involving a 24-year-old unmarried woman with both mature ovarian teratoma and YST within a brief 1-year interval. Regular reexamination protocols facilitated the early-stage detection of YST. The patient underwent surgical treatment, chemotherapy, and measures to preserve ovarian function, resulting in a favorable prognosis. Our primary purpose is to distill clinical insights from the diagnostic and therapeutic journey of this patient. Our purpose is to enhance medical professionals’ awareness that YST may be secondary to mature teratoma. Additionally, we underscore the critical importance of routine postoperative surveillance for ovarian mature teratoma, emphasizing its pivotal role in early malignant tumor detection—a factor paramount to the prognosis of patients.

## Introduction

Approximately 90% of ovarian cancers manifest as epithelial cell types, presenting a diverse array of histological variations. Conversely, the remaining 10% comprise non-epithelial ovarian cancers, which include a majority of germ cell tumors, sex cord-stromal tumors, and a subset of exceedingly rare entities such as small cell carcinomas. Ovary germ cell tumors are the most common ovarian neoplasms in women until 30 years of age, originating from germ cells. With the exception of certain tissue types, such as mature teratoma, the majority of ovarian germ cell tumors are malignant and are often diagnosed at the early stage (60%–70%) (1). Ovarian mature teratomas are the most common ovarian germ cell tumor (2), accounting for 10%–20% of ovarian tumors (3). Typically, these teratomas measure 5–10 cm, with only approximately 9% exceeding 15 cm (2). Malignant ovarian germ cell tumors (MOGCTs) are thought to originate from primordial germ cells, featuring inherited or somatic acquired alterations (4). MOGCTs accounts for 5% of ovarian germ cell tumors (5), principally in the teenage years. The pathogenesis of malignant ovarian germ cell tumors remains elusive, potentially related to genetic or environmental factors. Surgery or surgery combined with chemoradiotherapy can significantly improve the prognosis (6). Among MOGCTs, YST accounts for 14%–20% of ovarian malignant germ cell tumors (7), and the incidence is approximately 0.048/100,000 (8). YST is highly malignant and easy to early metastasize and relapse. YSTs often present challenges in treatment due to chemotherapy resistance upon recurrence. We have conducted extensive literature reviews. There are many studies on the secondary occurrence of YST in immature teratoma (9–12).

However, the occurrence of YST subsequent to mature teratoma surgery is rare. In the literature that we searched, there were no documented instances of secondary YST after ovarian mature teratoma surgery. We report a case of giant ovarian YST only 1 year after surgery for mature ovarian teratoma, accompanied by a comprehensive review of pertinent literature. The diagnosis and treatment of this patient are meticulously summarized and analyzed. The patient underwent both surgery and chemotherapy, with a favorable prognosis attributed to the early detection of the YST.

## Case presentation

The patient, a 24-year-old unmarried woman with no sexual history, sought medical attention at our hospital in August 2019 following the discovery of a pelvic cyst during a physical examination. 3D transrectal ultrasonography showed a mixed echo measuring 128 mm × 111 mm × 109 mm at the right anterior quadrant of the uterus. Before the operation, pelvic magnetic resonance imaging (MRI) indicated a large solid cystic mixed-signal mass in the pelvis, exhibiting well-defined boundaries and measuring a maximum cross-section of 158.56 mm × 83.74 mm × 112.54 mm. The mass exhibited closely proximity to the right ovary. The right ovarian mature teratoma was removed by conducting a single-hole laparoscopic operation at our hospital. During the operation, no significant abnormalities were found in the left ovary and bilateral fallopian tubes. The postoperative pathology was mature cystic teratoma. The patient recovered well from the operation.

In October 2020, a routine physical examination revealed a left adnexa cyst in the patient with a maximum diameter of 3 cm. Then, the color ultrasound at our hospital identified an echoless area measuring approximately 35 mm × 14 mm in the left accessory region. By January 2021, a follow-up color ultrasound of our hospital exhibited a cystic mass with a range of 104 mm × 105 mm × 68mm in the left ovary, with a high echo approximately 41 mm × 33 mm in the mass. The level of α-fetoprotein (AFP) was 72.20 IU/mL. A pelvic MRI showed a vast solid cystic mass in the pelvis, featuring clumpy solid components within the capsule. The mass measured approximately 112 mm × 78 mm × 128 mm and exerted compression on the adjacent uterus, bowel duct, and the right oviduct and ovary, encapsulated in its entirety.

Because the patient had not yet married and had not given birth, both the patient and her parents expressed a strong desire to preserve her fertility function as much as possible. Considering the potential malignancy of the ovarian tumor, we discussed fertility preservation for the patient and made a plan with the Department of Pathology and the Reproductive Center before the operation. Ultrasound showed that the dominant follicle was in the right ovary, with an average number of follicles observed in both ovaries. The patient’s Anti-Müllerian Hormone (AMH) level measured 34.04 pmol/L, indicating a robust reserve function in both ovaries.

We performed an exploratory laparotomy, during which the left ovary exhibited a significant enlargement, measuring approximately 12 cm × 12 cm × 10 cm, with a smooth surface. The appearance of bilateral fallopian tubes and the right ovary was normal. There was approximately 50 ml of dark red bloody effusion in the pelvic cavity, with no abnormalities noted in the peritoneal of the pelvic abdominal cavity. A thorough examination of various surfaces, including the liver, spleen, ligaments, diaphragm, large omentum, and intestinal tubes, revealed no apparent abnormalities. A total of approximately 1,000 mL of dark red translucent liquid was aspirated from the left ovarian cyst, and the left ovarian cysts were completely excised. The yellow irregular meat-like tissue, approximately 4 cm × 4 cm, was identified in the ovarian cyst cavity. The intraoperative pathological analysis confirmed the presence of an ovarian YST. After consulting with the patient’s parent, the left fallopian tube ovary and large omentum were removed, and a multi-point biopsy on the pelvic peritoneum was conducted. The right ovarian cortex, approximately 1 cm × 1 cm, was excised for cryopreservation of the ovarian tissue. Simultaneously, the reproductive physician cryopreserved the oocytes from the left ovary.

The postoperative pathological examination was a yolk cystic tumor. The postoperative diagnosis was a stage IC left ovarian yolk sac tumor. After surgery, she underwent chemotherapy with a combination of bleomycin, etoposide, and cisplatin for four cycles. Gonadotropin-releasing hormone agonist (GnRH-a) was administered to protect her ovarian function throughout chemotherapy with a total of four administrations, spaced at 28-day intervals. AFP was normal after the first cycle of chemotherapy. She resumed menstruating more than 3 months after the last chemotherapy treatment. There has been no recurrence of the disease for 33 months follow-up to now.

## Discussion

The occurrence of ovarian YST after the operation of ovarian mature teratoma is exceptionally rare. In this case, the patient sequentially developed ovarian mature teratoma and ovarian YST within only a 1-year interval. It has been documented that some non-ovarian YSTs are secondary to teratoma. We conducted extensive literature searches to gather clinical characteristics, summarizing them in **Table 1**. Yoshida’s study revealed that the rate of YST developing after sacrococcygeal teratoma in children was 5.4%, while there was no statistically significant difference in the incidence of secondary to mature teratoma or immature teratoma (5.2% vs. 6.4%) (14). The possible mechanisms of recurrent YST after the surgery of mature teratoma are outlined below. First, yolk sac tumor is considered as malignant transformation of teratoma (18, 19), and mature teratoma is malignant prelesion (16). The possibility of subsequent malignant development exists even for mature teratoma (17). Second, YST lesions may be microscopic and often cannot be positive for AFP, so they are easily ignored (20). Some researchers believe the YST develops from undetected small yolk sac lesions in the original teratoma (18, 21). Both views are considered to be forms of teratoma recurrence, regardless of the presence or absence of YST lesions in the primary teratoma. YST has a variety of histological patterns, and it is difficult to identify specific subtypes. Therefore, when the pathological specimen is a mature teratoma, it is necessary to thoroughly sample the tumor and carefully examine the pathological section to identify the malignant tumor components (22, 23). Third, Yoshida et al. hold a different perspective, suggesting that teratoma and secondary YST are metachronous multifocal germ cell tumors, which are the presence of multiple *de novo* tumors arising in different sites after long intervals, rather than the transformation of residual teratoma (14). AFP level plays a crucial role in the early diagnosis of YST and monitoring treatment effect (24). Even in postmenopausal patients with ovarian tumors, AFP is recommended to be tested for early detection of ovarian malignant germ cell tumors (25). It is recommended that patients with sacrococcygeal teratoma should be tested for AFP every 3 months for 3 years after surgery to facilitate early detection of YST (14). Activation and malignant transformation of mature teratoma may occur after 7 years (15). Serum AFP concentration above 100 ng/dL almost always indicates the presence of a YST focus. However, the detection of AFP is not emphasized in the clinical periodic review of ovarian mature teratoma (20). In this case, if AFP was detected at the same time when the left ovarian tumor volume was 3 cm, the yolk sac tumor would possibly be detected earlier. While the existence of ovarian YST alongside the previous ovarian mature teratoma may be a random occurrence, further research is warranted on the correlation between mature ovarian teratoma and YST.

**Table 1:** Clinical features of reported cases of secondary YST after mature teratoma.

Author (year)	Age, Sex	Symptom	Initial tumor location, and diagnosis	Secondary tumor (YST) location	Adjuvant therapy	Prognosis	Time interval between initial and secondary tumor
Rahadiani N (13) (2019)	2-day-old infant, female	dyspnea	buccal mucosa, mature teratoma	around the site of previous surgery scar	no	/	16 months
Yoshida M (14) (2013)	mean age: 7.1 months. female and male	/	sacrococcygeal, mature teratoma(9 cases), immature teratoma (4 cases)	sacrococcygeal	platinum-based chemotherapy, or radiotherapy after chemotherapy	follow-up time: 1–24 years. 11 patients without recurrence; 2 patients died at 2 and 4 years after the diagnosis of YST.	5–30 months
Utsuki S (15) (2007)	9-year-old, male	anorexia, nausea, and vomiting without headache	intracranial, mature teratoma	in the third ventricle	chemotherapy: cisplatin + etoposide, local irradiation and spinal irradiation	died 15 months after the second hospitalization	7 years
Ohno Y (16) (1998)	18-month-old, female	an expanding abdominal girth	right retroperitoneal, a mature cystic teratoma with an area of endodermal sinus tumor differentiation	/	/	no recurrence at 50 months of age	/
Byard R W (17) (1991)	neonatus, female	a large polypoid mass	Nasopharynx, mature teratoma	nasopharynx	chemotherapy: doxorubicin hydrochloride (Adriamycin)+ cyclophosphamide + dactimomycin (Actinomycin D) + vincristine and local radiation	recurrence after 16 months, died 18 months after recurrence	3 years

/, not available.

After surgery, mature ovarian teratoma may recur in the ipsilateral or contralateral ovaries. The intermediate and long-term recurrence rate in one study was 4.2%. The risk of recurrence increases if mature ovarian teratoma is bilateral, multiple, above 8 cm in diameter, with bone and central nervous system components (26, 27). Various tissue components of mature teratoma will have a secondary malignant transformation potentiality, a phenomenon more common in postmenopausal women. The malignant transformation rate is approximately 0.17%–2%, with 80% developing into squamous cell carcinoma, carrying a poor prognosis (28). Overexpression of p53, incomplete tumor resection, and tumor grade are risk factors for ovarian teratoma recurrence (29). In this case, the right ovarian mature teratoma was removed in 2019. The tumor was sizable, and the operation was performed using transumbilical single-incision laparoscopy, heightening the risk of ovarian cyst rupture (30). After the rupture of the tumor capsule wall, even a large amount of irrigation for the abdominal cavity could not avoid the residue of the contents, which increased the possibility of secondary malignant tumors (31). For giant teratoma, pathological examination is particularly challenging for the detection of malignant tumor components. Therefore, intraoperative rupture of tumor components should be minimized even for benign ovarian tumors to avoid tumor residue.

YST is highly malignant, prone to metastasize in the early stage, and carries a poor prognosis upon recurrence. Due to the absence of specific diagnostic markers, it is difficult to diagnose before surgery. In this case, the patient underwent regular color ultrasound after the operation of the ovarian mature teratoma. She promptly consulted a doctor when the ovarian cyst was detected so that the yolk sac tumor could be detected at an early stage without metastasis. In this case, ultrasound examination at an interval of 3 months showed that the ovarian tumor increased rapidly, threefold the ovarian tumor’s initial volume. According to imaging examination and clinical characteristics, the possibility of malignancy could not be ruled out. For patients with ovarian tumors with rapid growth or large tumor volume in a short period, it may be caused by internal tumor bleeding or tissue necrosis (28), and physicians should be vigilant about whether it is a malignant tumor.

The preferred treatment option is surgery combined with chemotherapy for YST. As we know, high-grade serous ovarian cancer (HGSOC), constituting 75% of epithelial ovarian cancers, is highly chemosensitive, primarily characterized by uniform *TP53* mutants (32). While ovarian YSTs rarely exhibit *TP53* mutation (33), unlike HGSOC, they still demonstrate sensitivity to chemotherapy. Therefore, ovarian YST is sensitive to chemotherapy. Postoperative chemotherapy is recommended for all stages of ovarian YST to improve prognosis (34), and fertility preservation surgery is feasible regardless of stage. The thoroughness of initial surgical treatment and postoperative chemotherapy are independent risk factors for progression-free survival (35). Various factors, such as the malignant tumor itself, ovarian surgery, pelvic surgery, hyperthermic intraperitoneal chemotherapy, and chemoradiotherapy, may lead to a reduction in ovarian reserve function (36, 37). For MOGCTs, the fertility-sparing comprehensive surgical staging includes the excision of the affected unilateral salpingo-oophorectomy, preservation of the uterus and the contralateral ovary, or preservation of one or both normal ovarian tissues and uterus if both ovaries are involved, in addition to biopsy or excision of the omentum, and excision of lymph nodes depending on age and stage. Considering that YST is more common in children and young women, fertility-sparing surgery to preserve and protect fertility is particularly important for patients’ quality of life in the future (38, 39). For unilateral malignant germ cell tumors, even in an advanced stage, fertility preservation surgery can be performed (40). The patient was 24 years old and unmarried in the case, and corresponding measures were taken to protect ovarian function before, during, and after surgery.

For women with fertility requirements, multidisciplinary consultation can be conducted before surgery to reduce the misdiagnosis rate and avoid over-treatment. Hormone levels and color ultrasound can determine initial ovarian reserve function. During the operation, immature oocytes can be extracted from the ovary for cryopreservation (41), and ovarian cortex cryopreservation can be performed (42). Frozen ovarian tissues can be used to isolate follicles or for transplantation. Resuscitation transplantation of cryopreserved ovarian tissue can increase autologous hormone levels and the probability of natural pregnancy. However, there may be a potential risk of malignant tumor cells implantation during transplantation, although this risk is low in other malignant tumors except in leukemia patients (43). For post-pubertal patients and patients with delayed chemoradiotherapy, it is feasible to obtain mature oocytes by promoting ovulation after surgery. Immature oocyte cryopreservation can be performed in preadolescent girls and patients with hormone-sensitive tumors (44).

Given the numerous previous studies on the protection of ovarian function by GnRH-a, the patient in this case received GnRH-a to protect ovarian function before chemotherapy. The GnRH-a inhibits ovarian function, prevents ovarian follicle recruitment, and prevents follicle growth and ovulation. The GnRH-a suppresses the secretion of endogenous gonadotropic hormone and follicle-stimulating hormone (FSH) levels and puts the follicle cells in a dormant state (45, 46). Therefore, reducing the sensitivity of follicles to chemotherapy drugs can minimize the destruction of follicles induced by chemotherapy and reduce the accumulation of chemotherapy drugs in ovarian tissue to reduce the damage to the ovary during chemotherapy. However, the role of GnRH-a in protecting ovarian function in patients with malignant tumors remains controversial (47–49). In this case, appropriate measures were taken to protect the patient’s fertility before, during, and after the operation. The diagnostic and treatment process serves as a valuable learning experience for clinicians.

## Conclusion

The genomic landscape and pathogenesis of ovarian YST remain elusive, posing challenges to study the diagnosis, treatment, and prognosis of the disease. The relationship between the occurrence of YST and ovarian mature teratoma cannot be determined. Because YST may be secondary to teratoma, patients with ovarian tumor after treatment of ovarian teratoma need to be vigilant about the possibility of YST. Attention should be paid to whether laparoscopic surgery increases the residual tumor lesions, especially single-incision laparoscopic surgery. Clinical surgeons are urged to minimize the exposure of tumor contents to the abdominal cavity during mature teratoma operations. Both physicians and patients should prioritize adherence to medical advice and engage in regular postoperative follow-up examinations, even for mature teratoma. These measures are paramount for the timely detection of ovarian malignant tumors, emphasizing the collaborative role of medical professionals and patients in ensuring comprehensive postoperative care.

## Data availability statement

The original contributions presented in the study are included in the article/supplementary material. Further inquiries can be directed to the corresponding authors.

## Ethics statement

The studies involving humans were approved by the Ethics Committee of the Third Affiliated Hospital of Zhengzhou University. The studies were conducted in accordance with the local legislation and institutional requirements. The participants provided their written informed consent to participate in this study. Written informed consent was obtained from the individual(s) for the publication of any potentially identifiable images or data included in this article. Written informed consent was obtained from the participant/patient(s) for the publication of this case report.

## Author contributions

SL: Data curation, Investigation, Writing – original draft. JP: Data curation, Formal analysis, Writing – review & editing. YZ: Data curation, Investigation, Writing – review & editing. DL: Data curation, Writing – review & editing. LL: Conceptualization, Supervision, Writing – review & editing. MN: Conceptualization, Supervision, Writing – review & editing.
